# The Role of Oxidative Stress and Inflammation in X-Link Adrenoleukodystrophy

**DOI:** 10.3389/fnut.2022.864358

**Published:** 2022-04-08

**Authors:** Jiayu Yu, Ting Chen, Xin Guo, Mohammad Ishraq Zafar, Huiqing Li, Zhihua Wang, Juan Zheng

**Affiliations:** ^1^Department of Endocrinology, Union Hospital, Tongji Medical College, Huazhong University of Science and Technology, Wuhan, China; ^2^Hubei Provincial Clinical Research Center for Diabetes and Metabolic Disorders, Wuhan, China; ^3^Department of Nutrition and Food Hygiene, School of Public Health, Cheeloo College of Medicine, Shandong University, Jinan, China; ^4^Institute of Reproductive Health/Center of Reproductive Medicine, Tongji Medical College, Huazhong University of Science and Technology, Wuhan, China

**Keywords:** X-ALD, oxidative stress, inflammation, biomarker, pathogenesis, treatment

## Abstract

X-linked adrenoleukodystrophy (X-ALD) is an inherited disease caused by a mutation in the ABCD1 gene encoding a peroxisomal transmembrane protein. It is characterized by the accumulation of very-long-chain fatty acids (VLCFAs) in body fluids and tissues, leading to progressive demyelination and adrenal insufficiency. ALD has various phenotypes, among which the most common and severe is childhood cerebral adrenoleukodystrophy (CCALD). The pathophysiological mechanisms of ALD remain unclear, but some *in vitro/in vivo* research showed that VLCFA could induce oxidative stress and inflammation, leading to damage. In addition, the evidence that oxidative stress and inflammation are increased in patients with X-ALD also proves that it is a potential mechanism of brain and adrenal damage. Therefore, normalizing the redox balance becomes a critical therapeutic target. This study focuses on the possible predictors of the severity and progression of X-ALD, the potential mechanisms of pathogenesis, and the promising targeted drugs involved in oxidative stress and inflammation.

## Introduction

X-linked adrenoleukodystrophy (X-ALD) is the most common peroxisomal disorder with an estimated incidence ratio of 1:14,700 in neonates (1), caused by mutations in the ABCD1 gene. Until present, over 2,700 types of mutations have been identified (2). ABCD1 codes the protein of adenosine 5′-triphosphate (ATP) binding cassette subfamily D member 1, which is located in the peroxisomal membrane to transport the very-long-chain fatty acids (VLCFAs) into peroxisomes for β-oxidation (3). The dysfunctional ABCD1 causes the failure of VLCFA degradation (4, 5), leading to the accumulation of VLCFA, especially C:26 and C:24 in tissues and plasma, and damage to organs, particularly in the cerebral white matter, spinal cord, and adrenal cortex (6, 7). Thus, VLCFA constitutes pathognomonic biomarkers for X-ALD diagnosis (8).

Various phenotypes are presented in X-ALD, including cerebral ALD (CALD), adrenomyeloneuropathy (AMN), Addison-only (AO), and presymptomatic ALD, among which the most severe is CALD (9, 10). Approximately one-third of boys with X-ALD develop CALD under the age of 12 years (11), characterized by initial learning and behavioral problems, followed by a rapid and severe progressive inflammatory demyelination resulting in a severe cognitive and physical disorder with a total disability that develops within 6 months to 2 years and dies within 5–10 years of the diagnosis (12–14). Besides, AMN, which is another prominent clinical phenotype of X-ALD that mainly manifests in adults, develops progressive stiffness and weakness of the legs, an impaired sense of vibration, and sphincter disturbances. The risk of developing CALD secondary to AMN is estimated to be at least 20% over 10 years (15). While other phenotypes are also likely to develop to cerebral form, the exact risk is unclear, and the occurrence of CALD remains unpredictable. Therefore, the identification of reliable biomarkers is of utmost importance, and it is critically required to predict the occurrence of disease and progression. Several countries have approved the newborn screening program of X-ALD in addition to routine screening (16–18).

However, the pathogenesis mechanism of X-ALD remains obscure. Oxidation stress and inflammation are the most typical features in most neurodegenerative diseases, including Alzheimer’s disease (AD), Parkinson’s disease (PD), and multiple sclerosis (MS) (19–21). Therefore, it is believed that oxidation damage and inflammation are the main pathogenesis of neuropathy, which are also found in patients with X-ALD (22–24).

Oxidative stress is “a transient or long-term increase in steady-state reactive oxygen species (ROS) levels, disturbing cellular metabolic and signaling pathways, particularly ROS-based ones, and leading to oxidative modifications of an organism’s macromolecules that, if not counterbalanced, may culminate in cell death *via* necrosis or apoptosis” (25). ROS includes both free radicals such as O^2–^, OH^–^, and more stable peroxide molecules such as H_2_O_2_ (26, 27). Because of the very short half-life and rapid reactivity of ROS, currently, there is no reliable method or technology to measure its level. Consequently, redox levels are mostly indirectly reflected by measuring antioxidant capacity (28).

Antioxidant defense systems include enzymatic and non-enzymatic types. The enzymatic antioxidant system predominately comprises superoxide dismutase (SOD), catalase (CAT), and glutathione peroxidase (GPX), and the non-enzymatic antioxidant system is classified as endogenous and exogenous antioxidants, the former including GSH, uric acid, transferrin, and the latter encompassing parts of vitamins, α-tocopherol, α-lipoic acid, and others (22). NRF2 is the master regulator of the endogenous antioxidant system, and ROS activate the NRF2 pathway to promote the transcription of downstream genes including heme oxygenase-1 (HO-1), glutamate-cysteine ligase (GCL), and NAD(P)H: quinone oxidoreductase-1 (NQO1), subsequently regulating the metabolism of endogenous antioxidants to defense oxidative stress (29, 30). The antioxidant system maintains redox balance *in vivo*, but oxidative stress occurs if it is insufficient to resist excessive ROS. Moreover, ROS directly stimulates the release of pro-inflammatory cytokines and activates the nuclear factor κB (NF-κB) pathway to cause inflammation (31, 32).

In the three-hit hypothesis of X-ALD, oxidative stress is described as the first hit to cause axonal injury of AMN, or subsequently induce inflammatory demyelination and cell death of CALD, triggered by stochastic factors such as lipid peroxides, environmental factors, and genetics (33). The exact cause of the onset and damage caused by oxidative stress and inflammation in X-ALD was unknown. However multiple *in vivo*/*in vitro* studies have demonstrated that drugs targeting redox imbalance and inflammation effectively improve symptoms and prevent disease progression (8, 34–37).

This study introduces the relationship between the X-ALD and oxidative stress and inflammation. Moreover, we will discuss the possible predictors for the severity and progression of X-ALD, the potential mechanisms of pathogenesis, and the promising targeted drugs involved in oxidative stress and inflammation.

## Increased Oxidative Stress and Inflammation in the Brain and Adrenal Gland

ROS in cells is mainly produced by mitochondria. The brain white matter and adrenal gland, which are involved in X-ALD, are high metabolic tissues with active mitochondria (38). In addition, both the brain and adrenal glands are lipid-rich organs that are highly susceptible to oxidative stress and have active cholesterol metabolism (39). Cholesterol metabolism is closely related to redox balance because mitochondrial ROS is produced during cholesterol generation; conversely, oxidative stress can obstruct steroidogenesis (40). Thus, oxidative stress may be the underlying influencing factor that contributes to predisposed X-ALD sites.

As the vital organ in our body, the brain consumes approximately 20% of total oxygen (41). High oxygen consumption tends to be associated with high utilization of mitochondria in the brain (30, 42, 43), leading to ROS production. In addition, axons and myelin in the white matter have an extremely high energy demand, which is mostly met by mitochondria to support the function of neural conduction. However, the brain’s antioxidant capacity is frail (41, 44, 45). As a result, hyperactive mitochondrial metabolism and a fragile antioxidant defense system may lead to the white matter susceptibility to oxidative stress.

Many oxidative markers such as manganese SOD (MnSOD), HO-1, lipid peroxidation 4-hydroxynonenal (4-HNE), malondialdehyde (MAL), and oxidized proteins (protein carbonyl) were found in the postmortem brains of patients with AMN and CALD (46–48), and the immunoreactivities of these indexes are related to the degree of inflammation and myelin destruction (46). Plasmalogen synthesized by peroxisome, an indicator of peroxisome disease diagnosis, is an important lipophilic myelin endogenous antioxidant that protects neural integrity and functions against oxidative stress (49). Researchers have found plasmalogens were reduced in the brain white matter of patients with CALD, and the reduction was associated with increased oxidative stress (48). Besides, recent studies also exhibit that the expression of various heat shock proteins (HSPs) increases in the brain tissue of X-ALD, while HSPs are upregulated after oxidative stress, which triggers the release of pro-inflammatory factors (49, 50). Furthermore, this increase in HSPs was observed before inflammation and demyelination, suggesting that oxidative stress as an early injury may be involved in the pathogenesis of CALD.

Inflammation in the brain is an essential characteristic for distinguishing the severity of X-ALD. CALD is a neuroinflammatory disease with many pro-inflammatory macrophages/microglia in the area of the lesion (51, 52), which was not found in AMN. The expression of tumor necrosis factor α (TNF-α) and other pro-inflammatory mediators was found to be increased in the demyelinated regions of patients with CALD (24, 53–55). The different cytokine expressions inferred that the lesion mainly involved a proinflammatory T-helper type 1 (Th1) response (56–58). Interferon γ (IFN-γ) is a specific effector cytokine of the Th1 response that could lead to oxidative damage through the production of reactive species, which is high immunoactivity in affected areas of CLAD while almost non-reactive in the ALD heterozygote and patients with AMN (46).

However, unlike the brain, the adrenal gland has a robust antioxidant capacity (59), so oxidative stress in the adrenal gland may be related to a high turnover of lipids and ROS production in steroidogenesis. Although studies have found significant markers of oxidative stress in the adrenal cortex of X-ALD model mice, there was no exact change in the adrenal glands of patients with X-ALD (46, 60). As steroidogenesis is heavily influenced by mitochondrial function and redox homeostasis, a redox imbalance in the adrenal gland may suppress cortisol production, resulting in adrenal insufficiency.

## Redox Imbalance and Inflammatory Markers in the Blood Reflect Disease Progression

There was no definite correlation between phenotypes and genotypes in X-ALD, and the symptoms of the disease varied considerably during the progression. Therefore, early diagnosis and progression prediction of the disease are necessary to improve the prognosis. Diagnosis of brain injury by magnetic resonance imaging (MRI) or clinical symptoms is extremely limited because most patients are already in an advanced stage of the disease; in some cases, inflammatory demyelination was found after autopsy. Some studies have used skin fibroblasts to predict disease severity by measuring levels of glycosphingolipid (GSL) species, reflecting more complex lipid metabolism (61). Researchers have even taken skin biopsies to extract and culture-induced pluripotent stem cells (iPSCs) to mimic brain tissue to get an early and accurate diagnosis of X-ALD (62). Compared with the high cost and hysteresis of MRI, and the invasiveness and harm of skin biopsy, blood-based biomarkers for X-ALD have gained prominence due to safety and convenience. Plasma VLCFA is a pathognomonic biomarker for X-ALD but independent of the severity of the disease. As important pathogenesis of X-ALD, oxidative stress and inflammation, the redox imbalance, and inflammatory indicators in blood could become biomarkers for disease prediction.

### Changes in Antioxidant Capacity and Peroxides in Different Phenotypes

Oxidative stress in the body is usually reflected by antioxidant capacity. SOD is a critical enzymatic antioxidant *in vivo*, which effectively dismutases the active superoxide anion into less reactive H_2_O_2_, thus eliminating excessive ROS. A study has shown that SOD activity and level in plasma are significantly different in healthy controls, heterozygote carriers, patients with AMN, and CALD, and are decreased with the severity of symptoms. Besides, plasma SOD level was negatively correlated with MRI severity in patients with CALD (63). The results suggest that SOD in plasma likely serves as a potential biomarker for disease progression and as a predictor for the onset and prognosis of cerebral damage. Another major endogenous antioxidant is GSH, which is sensitive to changes in redox balance (64). Additionally, GSH blood concentration is an important indicator of whole-body GSH status, especially in some hard-to-reach tissues such as the brain (22, 65). GSH imbalance was observed in whole blood, including lymphocytes, erythrocytes, monocytes, and plasma of patients with X-ALD. One study has demonstrated that the level of GSH in lymphocytes of patients with AMN was significantly lower compared to patients with CALD (66), but another study showed GSH levels in CALD monocyte were markedly decreased, but not in patients with AMN (67), which may be an indicator to distinguish AMN from CLAD. However, previous research also demonstrated that GSH concentrations in plasma are all significantly decreased in patients with X-ALD having any phenotype (such as AMN, AO, or CALD) (68). Therefore, whether GSH is a promising biomarker remains to be determined.

Markers of oxidative damage to DNA, proteins, and lipids in the plasma can also reflect the degree of oxidative stress and disease severity *in vivo*, especially lipid peroxidation. The levels of lipid peroxide were increased in both symptomatic and asymptomatic patients compared with healthy subjects and were significantly higher in the patients with AMN than in patients with CALD and asymptomatic patients (8, 69). Furthermore, a remarkable reduction of oxidative damage markers was revealed after treatment with high-dose oxidants (8).

As to other non-enzymatic or enzymatic antioxidants, some investigations showed that both α-tocopherol and docosahexaenoic acid were shown to be non-differentially reduced in all phenotypes (68). In addition, one study found an increase in GPX activity in erythrocytes (47), whereas another observed no difference (70). Overall, none of the above indexes showed significance in assessing the severity, progression, and prognosis of X-ALD.

The antioxidant system *in vivo* is always in a state of flux, which could be increased in response to continuous oxidative stress or depleted in reaction to excessive oxidative stress. In addition, antioxidant capacity varies from the degree of oxidative imbalance in the body. Several studies have found more severe oxidative stress and worse overall antioxidant capacity in AMN, which may be because oxidative stress as a direct injury factor leads to the occurrence of AMN. However, in the pathological mechanism of CALD, oxidative stress works as an early injury to trigger neuroinflammation under certain conditions, which lead to severe demyelination and brain damage, resulting in neurodegeneration at last. Hence, inflammation may be a marker of brain damage.

### Inflammatory Factors in the Prediction of Disease Progression

Cytokine levels in the plasma are generally low, and a dynamic balance exists between pro-inflammatory and anti-inflammatory factors. However, the balance could be broken by inflammation, oxidative stress, or cell injury during the disease process (71, 72). The peripheral blood monocyte/macrophage cells from asymptomatic patients and patients with AMN and CLAD were all observed to have pro-inflammatory skewing (52, 55). Levels of pro-inflammatory cytokines, such as interleukin 1 β (IL-1β), IL-2, and IL-8, and TNF-α in plasma are significantly increased in asymptomatic patients compared to symptomatic patients, which are positively related to plasma C26:0. Besides, in AMN, the levels of anti-inflammatory IL-4 and IL-5 were mainly increased, which was negatively correlated with the VLCFA level (57). However, in CALD, both pro-inflammatory and anti-inflammatory factors are within the normal range. In a follow-up study, the previously asymptomatic patients, after progressing to the cerebral phenotype, show reduced levels of cytokines (57). Hence, there may be a time required for upregulation of inflammatory mediators in plasma in X-ALD: actively expressed in asymptomatic patients or patients with AMN, whereas turned normal when developed into CALD. These suggest inflammatory mediators might be an early biomarker of cerebral injury and demyelination. Inflammation might be the potential cause that asymptomatic or AMN developing into cerebral form. Therefore, plasmatic cytokines should be regularly tested in asymptomatic patients and patients with AMN to predict disease progression from asymptomatic or AMN to the CLAD.

In addition, antioxidant therapy can also improve inflammation. Various plasmatic cytokines and inflammatory markers in patients with AMN were reduced after multi-antioxidant combination therapy, among which inflammatory markers, such as monocyte chemoattractant protein-1 (MCP1) and 15-hydroxyeicosatetraenoic acid (15-HETE), were significantly downregulated to become the promising predictors of response to treatment (8).

In brief, these redox imbalances and inflammatory markers observed in the blood may be potential predictors of X-ALD, providing a reliable basis for disease severity, progression, as well as therapeutic response. However, as whole blood is a complex mix of blood cells and plasma, longitudinal comparisons should be conducted in plasma, lymphocytes, monocytes, and erythrocytes of antioxidant and inflammatory levels to evaluate better and predict disease.

## The Underlying Mechanisms of Onset and Damage Involved in Oxidative Stress and Inflammation of X-Linked Adrenoleukodystrophy

**FIGURE 1:**
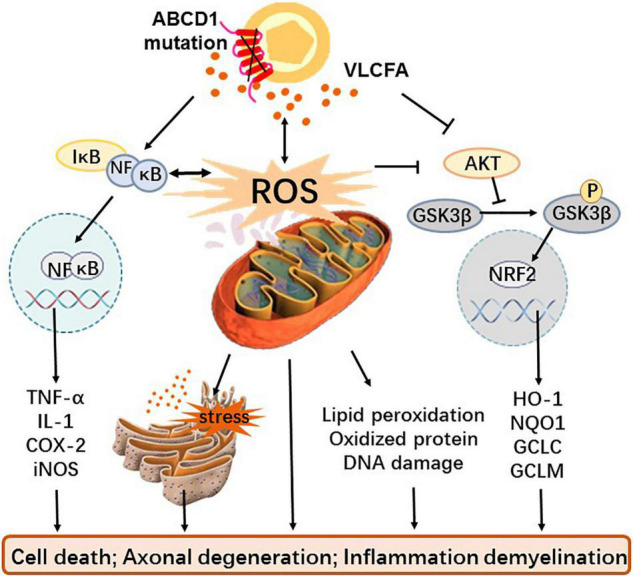
The role and mechanism of oxidative stress and inflammation in X-ALD. Mutations in the transmembrane protein ABCD1 gene on the peroxisome prevents VLCFA from being transported to the peroxisome for β-oxidation, resulting in the accumulation of VLCFA *in vivo*. Dysfunctional peroxisome and cumulative VLCFA not only causes mitochondria that give rise to excessive ROS and oxidative stress but also directly interferes with the GSK3β-NRF2 antioxidant pathway leading to redox imbalance and activate the NF-κB pathway to induce inflammation. In addition, excessive ROS can also affect antioxidant systems as well as inflammatory pathways, cause endoplasmic reticulum stress with or without VLCFA, and generate macromolecular peroxidation damage. These alterations eventually cause cell death, axonal degeneration, and even inflammatory demyelination.

Although oxidative stress and inflammation have been identified as important factors in the pathogenesis of X-ALD, neither the exact source of ROS and inflammatory factors nor the related signal pathway and molecular mechanism have been elucidated. Until present, excessive VLCFA has been considered the causative factor of oxidative stress and inflammation in X-ALD (73). Redox imbalance is caused by increased production of mitochondrial ROS due to excess of VLCFA and inadequate endogenous antioxidants; inflammation is a consequence of VLCFA directly or indirectly promoting the release of inflammatory factors (Figure 1).

In addition, various animal models with both advantages and drawbacks were used to study the mechanism of X-ALD (74, 75). ABCD1 knockout mice that exhibit delayed (20 months) onset axonopathy in the spinal cord without cerebral inflammatory demyelination (76), which are considered to mimic AMN, are often used to evaluate changes in biochemical markers. In addition, double mutant ABCD1/ABCD2 mice demonstrate earlier and even worse symptoms that are suitable for therapeutic assays at 12 months but also in the absence of inflammatory demyelination (77). Similarly, studies have shown that ABCD1 knockout or ABCD1/2 double-knockout rabbit models still fail to recapitulate human CALD phenotypes (78).

### The Cross-Talk Between Peroxisome and Mitochondria

Recently, the capacity of redox regulation of peroxisome has drawn great attention. Peroxisome plays a key role in cellular lipid metabolism and ROS, which not only generates ROS by itself (79) but also has interplay with mitochondria through messengers such as VLCFA, hydrogen peroxide, and PUFAs, leading to further oxidative stress (80, 81). Studies have revealed that inhibition of peroxisome activity or biogenesis rapidly results in mitochondrial oxidative damage and dysfunction (79). It is known that mitochondria are major regulators of redox. VLCFA may be the biological messengers that convey redox information between peroxisomes and mitochondria in X-ALD and then induce mitochondrial oxidative stress.

### Toxicity of Very-Long-Chain Fatty Acids

Until present, excessive VLCFA has been considered as a causative factor of oxidative stress and inflammation in X-ALD (73), although the role that VLCFA plays in the mechanism is indeterminate. Studies have shown that 20 μM or a higher dose of VLCFA can induce mitochondrial inner membrane depolarization and permeability transformation, resulting in excessive ROS, which leads to oxidative stress characterized by lipid peroxidation, protein carbonylation, increased SOD activity, decreased CAT activity, and glutathione level, especially on microglia, astrocytes, and adrenocortical cells (73, 79–83). ABCD1 deficiency can induce the spontaneous production of ROS in low doses or even in the absence of VLCFA. Studies have shown that in the concentration range of 5–10 μM, C:26 could significantly increase the level of ROS and decrease the level of GSH in ABCD1 gene knockout oligodendrocytes and X-ALD fibroblasts, and the oxidative damage products were doubled at high dose C:26 (79, 84).

Then, VLFCA can also directly regulate inflammation-related signal pathways to promote the increased expression of NF-κB and AP-1 (85), but another study suggests that VLCFA influences the precursors of inflammation-resolving lipid mediators to regulate inflammation response and oxidative stress (86). In addition, studies have demonstrated that dysregulation of VLCFA induces endoplasmic reticulum (ER) stress and lysosomal and peroxisomal dysfunctions (87). In summary, VLCFA plays the role of cytotoxicity in various ways, leading to oxidative and inflammatory damage.

### Silent Information Regulator 1/Coactivator-1/Peroxisome Proliferation-Activated Receptor Pathway and Mitochondrial Deletion

As previously mentioned, mitochondria are the main organelles that produce ROS in the brain; thus, mitochondrial dysfunction is the main cause of oxidative stress. Reduced levels of mitochondrial proteins such as NADH-ubiquinol oxidoreductase (NDUFB8) and voltage-dependent anion channel (VDAC) were observed in the affected white matter of patients with X-ALD (88), suggesting that mitochondrial content or function is impaired in X-ALD. Peroxisome proliferation-activated receptor-gamma (PPAR-γ) coactivator-1 (PGC-1) is the dominant regulator of mitochondrial biogenesis, regulated by upstream factor sirtuin 1 (SIRT1) partly, by changing the content and/or activity of transcription regulators such as PPARα/β/γ, estrogen-related receptor-α (ERR), nuclear respiratory factor 1 (NRF1), and the mitochondrial transcription factor (TFAM), which regulates the replication and transcription of mitochondrial DNA (88). The expression of SIRT1 in the affected cerebral white matter was lower than that in the unaffected white matter in the X-ALD human brain (89). Levels of SIRT1, PGC-1, and downstream transcription factors PPARα/β/γ, ERRα, and TFAM were all reported to decrease in the spinal cords of ABCD1-/- mice, further indicating impairment of mitochondrial biogenesis in the disease (88, 89).

In addition, the PPAR family, an important regulator, has been confirmed to demonstrate mitochondrial normalization and neuroprotection in various neurodegenerative diseases. Activation of PPARγ induces mitochondrial biogenesis and reverses energy depletion in X-ALD certainty. Erucic acid, an important ligand of PPARδ, has been used to treat X-ALD (90). The study showed that activation of PPARδ stimulated mitochondrial biogenesis and suppressed ROS production, protecting the brain from mitochondrial and oxidative damage (90). As for PPARα, it has been found that downregulation can affect antioxidant and anti-inflammatory responses in other degenerative diseases such as AD (91). However, in X-ALD, PPARα is not directly related to mitochondrial dysfunction and oxidative stress, but it may work by regulating the expression of ABCD1 homologous protein ABCD2 (77).

### Nuclear Factor E2-Related Factor and Antioxidative Systems

Nuclear factor E2-related factor (NRF2) is recognized as the master regulator of cellular redox homeostasis. Generally, NRF2 binds to Kelch-like ECH-associated protein (KEAP1) in the cytoplasm but then dissociates from KEAP1 and translocates into the nucleus where it combines with the antioxidant response element (ARE) in DNA sequence to activate the transcription of various downstream genes coded endogenous antioxidants, including GSH, SOD, and CAT under oxidative stress conditions, consequently starting the antioxidant defense systems (92). Activation of the KEAP1/NRF2 pathway blocks the progression of degenerative diseases such as AD and amyotrophic lateral sclerosis (ALS) (93, 94). There is no difference in the expression of NRF2 protein in total skin-derived fibroblasts between patients with X-ALD and healthy people, but the level of NRF2 located in the nucleus and transcription of downstream genes are both reduced (29). However, no study shows the interaction between KEAP1 and NRF2, or the expression of KEAP1 was changed in X-ALD. Conversely, the KEAP1-independent regulation of the AKT/GSK3β/NRF2 pathway may regulate the antioxidant system. Glycogen synthase kinase-3β (GSK3β), inhibited by AKT-mediated phosphorylation, is a protein kinase that can inhibit the NRF2 pathway by inducing NRF2 phosphorylation. Defective AKT phosphorylation and abnormal activation of GSK3β are both found in skin-derived fibroblasts from patients with X-ALD and in the spinal cord of ABCD1 knockout mice. Furthermore, when drugs inhibit GSK3β in X-ALD fibroblasts, NFR2 restores and activates transcription of downstream genes (29).

A variety of lipids produced by peroxisomes such as plasmalogen and DHA are also used as endogenous antioxidants to eliminate intercellular ROS to protect cells from oxidative damage (95). The genetic inactivation of PEX7 leads to defects in plasmalogen biosynthesis. It was found that PEX7:ABCD1 DKO mice showed more severe neuropathy with demyelination and axonal loss than ABCD1 KO mice (96), suggesting that plasmalogen plays an important role in the onset and development of X-ALD. The upregulation of NRF2 significantly reversed the decreased expression of plasmalogen in the AD model mouse brain to improve oxidative stress and neuroinflammation (97). Therefore, NRF2 may directly or indirectly regulate lipophilic antioxidants to play a role in redox regulation.

### Nuclear Factor κB Pathway and Inflammation

NF-κB is a redox-regulated transcription factors involving various responses such as pro-inflammation, inflammation, and oxidative stress (98). Typically, it is held in resting-state thought association with inhibitor of κB (IκB) proteins. When stimulants, including ROS, cytokines, and VLCFA, bind to cell surface receptor toll-like receptors (TLRs), IκB is degraded, while NF-κB is activated and then migrated into nuclear, NF-κb combines with DNA responsive elements to promote transcription of genes of pro-inflammation and inflammation (92). Activation of cerebral inflammation in X-ALD disease may be associated with the NF-κB pathway. The increased expression of TLR2 was 1.3-fold, and the TLR4 coreceptor CD14 was 4.5-fold higher in postmortem brain tissues of patients with CALD than in the control group (24), indicating that the TLRs/NF-κB pathway is activated. NF-κB was observed to have a significantly increased activation and DNA binding activity in primary astrocytes after ABCD1/ABCD2 silencing (85). In addition, in spinal cords of ABCD1 KO mice, the massage RNA and protein products of NF-κB were also notably upregulated (24). The primary astrocytes and spinal cord mentioned in previous studies were detected increased levels of downstream pro-inflammatory mediators TNFα, IL-1β, C-C chemokine ligand 5 (CCL5), and cyclooxygenases-2 (COX-2), which could also confirm the activation of the NF-κB pathway (24, 85).

In addition, studies have shown that the blood–brain barrier (BBB) endothelial cell dysfunction in X-ALD is also related to the activation of the NF-κB signaling pathway (35). Because NF-κB combines and activates the transcription of adhesion molecules, leukocytes, and inflammatory cells can cross the BBB to the brain. The research found NF-κB expression increased in ABCD1 silenced human brain microvascular endothelial cells (HBMECs) (99). One of the characteristics of inflammatory demyelination in CALD is the increased BBB permeability to monocytes/macrophages (99). ABCD1-deficient primary monocytes from patients with X-ALD predispose for pro-inflammatory but unobtained to anti-inflammatory polarization activation, triggering an inflammatory response (55).

### Peroxidation Induces Further Cell Damages

Excessive ROS can also indirectly affect the onset and progression of X-ALD by oxidizing modified macromolecules such as lipids, proteins, and nucleic acids in cells to change their structure and function. These peroxidation products could exacerbate functional impairment of mitochondria and peroxisomes and further induce oxidative stress and inflammation, leading to cellular damage and death (8, 34, 69, 84, 100, 101).

The brain has abundant lipids but low antioxidant defense, consequently, lipid peroxidation increases, notably when oxidative stress occurs. 7-Ketocholesterol (7-KC), one of the common oxidation products of cholesterol, has been found at increased levels in the plasma of different phenotypes of patients with X-ALD (68). *In vitro*, 7-KC was observed to inhibit the expression of ABCD1 and homologs, including ABCD2 and ABCD3 in microglia and oligodendrocytes, resulting in peroxisome dysfunction, the root of disease occurrence. Besides, 7-KC also promotes oxidative stress, mitochondrial dysfunction, and neuroinflammation, which are significant characteristics of X-ALD (68, 102). Another oxysterol, 25-hydroxycholesterol (25-HC), and the cholesterol 25-hydroxylase (H25CH) were found to be significantly upregulated in iPSCs and primary fibroblasts of patients with X-ALD. The increased level in CALD was more significant in AMN, suggesting that the expression of 25-HC and C25CH are closely associated with phenotypic severity to become a new biomarker. Moreover, the study showed that 25HC could induce inflammasome activation *via* stimulating mitochondrial ROS to cause microglial recruitment, IL-1β release, and oligodendrocyte death, and consequently severe neuroinflammation and demyelination (103). Lipid peroxidation, especially oxysterol, mainly accumulates in the inflammatory demyelinating lesions and adrenal cortex, which are the major affected areas in X-ALD. Thus, until present, cholesterol metabolism is considered to be strongly interrelated with the pathogenesis of X-ALD. Moreover, as active oxides in the body, lipid peroxidation can also damage other macromolecules.

Protein is the main functional molecule in cells, but the structure and function can be changed when it is oxidized and subsequently degraded by ubiquitin-proteasomes. Dysfunctions of the ubiquitin-proteasome system were found in both ABCD1 null mouse model spinal cords and X-ALD fibroblasts, which speculated that oxidative stress and inflammation stimulated an increase in oxidized proteins that needed to be ubiquitinated, consequently, resulting in the inhibition of proteasome activity because of massive accumulation of polyubiquitinated proteins (104). Mitochondria are the main participants of oxidative stress, and their proteins are observed to be oxidized damaged in the X-ALD model, leading to the dysfunction of key enzymes in mitochondrial energy metabolism, such as α/β-ATP synthase, the trichloroacetic acid cycle enzymes malate dehydrogenase, and aconitase (105). In another example, cyclophilin D, an important component of mitochondrial permeability transition pore, was observed to be overexpressed and oxidatively modified when stimulated by oxidative stress in affected brain tissue and skin-derived fibroblasts from patients with X-ALD as well as the spinal cord of X-ALD mouse models. High level and/or increased carbonylation of cyclophilin D enhances the sensitivity of ABCD1-deficient cells to oxidative stress and then induce the opening of the mitochondrial permeability transition pore, leading to inner mitochondrial membrane depolarization and respiratory chain damage, and ultimately cell necrosis and apoptosis (106). Nevertheless, the inactivation of the proteasome leads to the failure in the elimination of oxidative mitochondrial proteins in a timely manner, then aggravating the toxicity of the damaged mitochondria and leading to more production and accumulation of ROS (104). Hence, the accumulation of oxidized proteins and the ubiquitin-proteasome inactivation further induces oxidative stress.

Excess ROS also leads to DNA oxidative damage, which, if not repaired, can lead to cell senescence and apoptosis. Significantly increased DNA damage was ascertained in leukocytes of symptomatic patients with X-ALD, and the researchers supposed that the damage might be caused by lipid peroxidation (105). Compared to nuclear DNA, mtDNA is more sensitive to oxidative stress because the mitochondrial genome is deficient in protection and closer to ROS. Increased mtDNA oxidation ratios were detected in the affected white matter areas, showing active demyelinating plaques of patients with X-ALD (107).

### Endoplasmic Reticulum Stress Motivated by Oxidative Stress

Endoplasmic reticulum stress, or the unfolded protein response (UPR), has a complicated interaction with oxidative stress (108). It is currently believed to be closely associated with various peroxidase diseases and degenerative diseases such as AD, PD, and ALS. Although the accumulation of misfolded proteins is wildly considered to drive UPR, oxidative stress and lipid dyshomeostasis are perceived as the main cause of ER stress in X-ALD (108, 109). The evidence of UPR activation is detected in the X-ALD mouse model as well as skin-derived fibroblasts and brain tissues from patients with X-ALD (108–110). Furthermore, antioxidant treatment reversed ER stress and normalized the expression of related pathway molecules, which manifested that redox imbalance promoted UPR activation (109). Eventually, ER stress gives rise to cell apoptosis.

### Other Potential Targets Regulate Redox and Inflammation in X-Linked Adrenozleukodystrophy

Bioenergy failure is one of the futures of X-ALD, and AMP-activated protein kinase (AMPK) plays an important role in controlling energy homeostasis. Deficiency of AMPK expression was found in white matter, fibroblasts, and lymphocytes from patients with X-ALD, especially cerebral form (37, 111). AMPK regulates mitochondrial biosynthesis and function as well as inflammatory response. In the mixed glial cells of ABCD1-knock-out mice, loss of AMPK induced mitochondrial dysfunction caused by reduced PGC-1 levels and spontaneous increased pro-inflammatory tendencies (111, 112). Inhibition of AMPK promotes pro-inflammatory gene expression, while stimulation with inflammatory cytokines contributes to dephosphorylation and inhibits AMPK (112). The vicious cycle of AMPK deletion and inflammation activation exacerbates inflammatory damage, which would be the potential mechanism of severe cerebral inflammatory demyelination because a greater reduction in AMPK was found in CALD than in AMN (112).

Receptor-interacting protein 140 (RIP140), a coregulator of nuclear receptors and other transcription factors, serves dual functions as a co-activator and a co-repressor. RIP140 interacts with PGC-1 to negatively regulate mitochondrial biogenesis and energy homeostasis and interacts with NF-κB to induce inflammatory activation. A study found that RIP140 was upregulated in specific tissues that affected cerebral white matter in patients with CALD and spinal cord in ABCD1 mouse models, which may be due to the redox imbalance and excessive ROS required for RIP140 induction. Genic-silenced RIP140 restored mitochondrial function and redox homeostasis and prevented inflammatory response in X-ALD mouse models (36). Thus, inhibition of RIP140 may be regarded as a potential therapeutic target for X-ALD.

In conclusion, although the exact mechanisms underlying oxidative stress and inflammation in X-ALD are ambiguous, several possible mechanisms have been proposed and gradually validated in various cells and animal models. These mechanisms can be identified as potential intervention targets for the treatment of X-ALD, and drugs aimed at the part of targets have been applied to preclinical research or even clinical research to explore more new directions for therapy.

## Novel Therapeutic Strategies Targeted Oxidative Stress and Inflammation

Current clinical treatments are only Lorenzo’s oil (LO) and bone marrow transplant (BMT) or hematopoietic stem cell transplantation (HSCT), but the therapeutic effects have limitations and uncertainty. Yet the promising lentiviral hematopoietic stem cell (HSC) gene therapy was halted because it induced abnormal bone marrow cells even myelodysplastic syndrome (MDS) (113, 114). Consequently, more new therapeutic strategies need to be explored. Since oxidative stress and inflammation have been identified as key factors in the early stage of X-ALD, normalizing redox balance, mitochondrial function, and inflammation are key approaches in treatment.

### Promising Clinical Trial of X-Linked Adrenoleukodystrophy

As previously noted, oxidative stress caused by excessive ROS and defective antioxidant defense is an essential pathological mechanism of X-ALD, so antioxidants are naturally considered to be applied in the disease. N-Acetylcysteine (NAC), a strong thiol-containing antioxidant, can improve survival in advanced patients with CALD as adjunctive therapy to HSCT, which targets to increase HO-1 expression (115, 116). *In vitro* studies found that NAC alone could reduce VLCFA-induced oxidized damage, mitochondrial dysfunction, and inflammation (83, 100, 107), while dendrimer-NAC, which was revealed to travel the BBB and located specifically for better bioavailability in other cerebral injury models, increased GSH levels and minified proinflammatory cytokines (67). Furthermore, NAC still alleviated the oxidative damage of cyclophilin D in X-ALD mouse models and reduced its expression when combined with α-lipoic acid to suppress mitochondrial dysfunction. To improve the effectiveness of antioxidants, the therapy of combination of multiple antioxidants has entered the stage of clinical trials. NAC, α-tocopherol, and α-lipoic acid, which are well-known antioxidants proved to be able to cross BBB and to achieve a neuroprotective effect, were shown to restrain oxidative stress and damage and reverse the axonal degeneration and dyskinesia in combination in the preclinical study (34). Phase II trials showed the improvement of various biomarkers and athletic ability in patients with AMN for a year treatment with a high dose of NAC, α-tocopherol, and α-lipoic acid in combination. In addition, by comparing the expression of oxidative stress and inflammatory markers before and after treatment, the study provides a series of candidate biomarkers to predict antioxidants treatment response, patient stratification, and disease progressions such as MCP1 and 15-HETE (8). Unfortunately, this clinical study focused mainly on the curative effect in patients with AMN but neglected to evaluate in CALD.

As an agonist of PPARγ, pioglitazone reverses mitochondrial depletion and bioenergy failure *via* the activation of the PGC-1/PPARγ pathway and reduces oxidized damage of DNA as well as proteins through antioxidative action, preventing the progression of axonal degeneration and locomotor deficits (88). Besides, leriglitazone, a newly developed PPARγ agonist, has been revealed better therapeutic efficacy than pioglitazone because it possesses superior BBB penetration, bioavailability, and safety profile. Leriglitazone not only improves redox balance and mitochondrial biogenesis, more importantly, but it also inhibits the activation of NF-κB to control inflammation and effectively decreases pro-inflammatory biomarkers in plasma and cerebrospinal fluid in healthy volunteers, which is very important for the treatment of severe cerebral form (35). Hence, leriglitazone has been registered in phase II clinical trial for the treatment of CCALD and received orphan drug designation in Europe and the United States and is expected to become the first oral drug for the therapy of X-ALD.

### Preclinical Studies of X-Linked Adrenoleukodystrophy for New Therapy

Studies on SIRT1 show evidence for its role in preventing oxidative stress and inflammation (117). Resveratrol, the most common SIRT1 activator, not only relieves the brain microvascular endothelial dysfunction and the permeability of the BBB to inflammatory cells caused by ABCD1 deletion through enhancing SIRT1 function to regulate NF-κB signal pathway (99) but also normalizes axonal degeneration and locomotor deficits *via* activation of SIRT1/PGC-1 pathway to promote mitochondrial biogenesis and function in ABCD1-/ABCD2-/- mice (89). Furthermore, resveratrol is a natural antioxidant that regulates antioxidant defense by eliminating excessive ROS in X-ALD fibroblasts (89).

Biotin is an essential cofactor for carboxylases to control energy metabolism and redox balance (118). In X-ALD mice models, treatment with a high dosage of biotin removes excessive ROS by inducing the endogenous antioxidant response of NRF2 and normalizes redox *in vivo via* recovering mitochondrial biogenesis and energy supplement to improve symptoms of dyskinesia and axon injury consequently. In addition, biotin could target lipid metabolism to cure X-ALD (119).

Targeting to NRF2 antioxidant pathway is a novel therapeutic strategy. Dimethyl-formamide (DMF), a classical activator of NRF2, has already been used to treat some neurodegenerative diseases (120), which has also expressed excellent antioxidant properties in X-ALD. DMF reactivated the expression of NRF2 and the transcription of classic target genes such as HO-1 and NQO1 to reverse oxidative damage. Besides, DMF can also prevent mitochondrial and bioenergy failure and an inflammatory imbalance to ultimately improve the clinical symptoms of X-ALD mice (29).

The ER stress inhibitor tauroursodeoxycholic acid (TUDCA) has been confirmed to effectively normalize several UPR responsive genes and ER stress sensors aberrantly regulated in ABCD1-mice and stop the progression of axonal and locomotor impairment in ABCD1/ABCD2 double knout out mice (109). Moreover, TUDCA, which has remarkably safe profiles and penetration ability through BBB that has been applied in clinical trials for degenerative diseases such as ALS and HD, is looking forward to being a potential candidate drug for X-ALD.

Metformin, the most common AMPK activator, was found to promote mitochondrial generation and function, increase ABCD2 expression, and lower VLCFA concentration in the fibroblasts derived from patients with AMN and CALD. Furthermore, a study has found metformin-induced AMPK, ABCD2, and mitochondrial complex subunit levels *in vivo* in ABCD1-KO mice (37). But the effect on symptoms of this disease was not explored.

Histone deacetylase (HDAC) inhibitor is proved to inhibit oxidative stress and inflammation in the brain to improve cerebral damage (121, 122) and interfere with pro-inflammatory skewing (51) as well as oxidative damage of proteins (123) in X-ALD. SAHA, the pan-HDAC inhibitor in clinical practice, has been found to restore mitochondrial integrity and function in ABCD1 silenced oligodendrocytes and astrocytes (124) and reduce the expression of proinflammatory cytokines, iNOS, as well as the activation of NF-κB in ABCD1/ABCD2-silenced mice primary astrocytes (125).

### Current Clinical Treatment’s Effects on Oxidative Stress and Inflammation

BMT, HSCT, and LO are currently the main clinical treatments for X-ALD. Lipid peroxidation and oxidized protein were significantly reduced in the plasma of patients with X-ALD treated with BMT or HSCT, suggesting that BMT can lower oxidative stress *in vivo* (126). In contrast, although erucic acid, the important component of LO, was revealed to have antioxidative and anti-neuroinflammatory effects in therapies of AD, HD, and MS (90, 127), the study showed that LO did not reduce oxidative stress and promote the antioxidant defense system in patients (70).

Studies have shown that lovastatin significantly inhibits the production of pro-inflammatory cytokines in astrocytes and microglia and normalizes the level of VLCFA in skin fibroblasts of X-ALD by increasing β-oxidation (128). Increased levels of antioxidant plasmalogens and decreased levels of ROS were detected in X-ALD mice treated with lovastatin (48). However, plasma VLCFA levels in patients treated with lovastatin decreased by only about 20%, which is still much higher than the normal level (129). Therefore, the clinical significance of lovastatin in the treatment of X-ALD needs to be further studied.

Most treatments are limited to spinal axonal lesions and early asymptomatic periods due to the lack of suitable animal models and are hardly effective for CALD or progressive conditions. Besides, the brain is the major affected site, so whether the drug penetrates the BBB also has to be considered. In conclusion, though the drugs mentioned above showed good efficacy in preclinical or clinical trials, many tests and a long time would be required to apply to clinical treatment.

## Conclusion

There is increasing evidence of oxidative damage and inflammation in patients with X-ALD, and these would be correlated with the onset or severity of symptoms, making oxidative stress or inflammation indicators possible as biomarkers to predict disease progression. In particular, plasma inflammatory markers can be used as early biomarkers of brain injury (57) and predictors of therapeutic response and disease progression (8). Current studies on plasma cytokines have mostly included asymptomatic patients and patients with AMN and CALD but have not considered patients with only adrenal insufficiency. Especially adrenal insufficiency is often the first manifestation of X-ALD, frequently before the onset of neurologic symptoms (130), so the disease progression of AO patients is what we urgently want to know.

Although the exact molecular mechanism of the ROS and inflammation process is still unclear, continuous studies have found that mitochondrial dysfunction, redox imbalance, bioenergy failure, and inflammatory activation are the main features of the X-ALD, which may act synergistically with other underlying mechanisms involved in the pathophysiology of this disease, such as ER stress, leading to body damage. Ferroptosis, a newly identified regulatory form of cell death, is associated with the etiopathogenesis of various degenerative diseases, such as AD and PD, and has achieved positive effects in clinical treatments (131, 132). The typical characteristics of ferroptosis, including mitochondrial damage, GSH deficiency, and ROS accumulation, have also been found in X-ALD (69, 102). Malondialdehyde (MDA), an important ferroptosis marker and lipid peroxidation (133), increased significantly in plasma of patients with X-ALD and was markedly reduced after treatment with BMT (126). These results indicate that ferroptosis may provide new insights into the pathological mechanism and promising treatment of X-ALD.

Many promising therapeutic approaches targeted at oxidative stress and inflammation are being tested in preclinical or clinical trials with significant improvements in outcomes. Nevertheless, for the most severe phenotype of CALD, its molecular mechanisms and interventions are challenging to explore further due to the absence of appropriate experimental models. Hopefully, changes in oxidative stress and inflammation in CALD could be further explored and more promising treatments attempted.

## Author Contributions

JY drafted the manuscript. TC looked up the articles and revised this manuscript. XG, MIZ, HL, ZW, and JZ reviewed the manuscript structure and ideas during the development of the article. All authors contributed to the article and approved the submitted version.

## Conflict of Interest

The authors declare that the research was conducted in the absence of any commercial or financial relationships that could be construed as a potential conflict of interest.

## Publisher’s Note

All claims expressed in this article are solely those of the authors and do not necessarily represent those of their affiliated organizations, or those of the publisher, the editors and the reviewers. Any product that may be evaluated in this article, or claim that may be made by its manufacturer, is not guaranteed or endorsed by the publisher.
